# HPLC Analysis and Risk Assessment of 15 Priority PAHs in Human Blood Serum of COPD Patient from Urban and Rural Areas, Iasi (Romania)

**DOI:** 10.3390/jpm13091290

**Published:** 2023-08-23

**Authors:** Ioana Buculei, Mona Elisabeta Dobrin, Daniela Matei, Ilie Onu, Ionel Bogdan Cioroiu, Bogdan Caba, Mădălina-Gabriela Postelnicu, Dragos-Horia Buhociu, Carmina Liana Musat, Radu Crisan-Dabija, Andrei Tudor Cernomaz, Antigona Carmen Trofor

**Affiliations:** 1 Doctoral School, Faculty of Medicine, University of Medicine and Pharmacy “Grigore T. Popa”, 700115 Iasi, Romania; 2 Clinical Hospital of Pulmonary Diseases, 700115 Iasi, Romania; 3 Department of Biomedical Sciences, Faculty of Medical Bioengineering, University of Medicine and Pharmacy “Grigore T. Popa”, 700115 Iasi, Romania; 4 Doctoral School of the Faculty of Chemical Engineering and Environmental Protection “Cristofor Simionescu”, Technical University “Gheorghe Asachi”, 700050 Iasi, Romania; 5 Romanian Academy, Iasi Branch, Research Center for Oenology, 700505 Iasi, Romania; 6 Department of Sports Games and Physical Education, Faculty of Physical Education and Sport, “Dunărea de Jos” University of Galați, 800008 Galati, Romania; 7 Doctoral School of Urban Planning, Ion Mincu’ University of Architecture and Urban Planning, 010014 Bucharest, Romania; 8 Department of Morphological and Functional Sciences, Faculty of Medicine and Pharmacy, “Dunărea de Jos” University of Galati, 800008 Galati, Romania; 9 Faculty of Medicine, University of Medicine and Pharmacy “Grigore T. Popa”, 700115 Iasi, Romania; 10 Pulmonology Department, Clinic of Pulmonary Diseases, 700115 Iasi, Romania

**Keywords:** air pollution, PAHs, COPD, chemical exposure, risk assessment

## Abstract

One of the leading risk factors for environmental health problems is air pollution. The World Health Organization (WHO) reports that this risk factor is associated with one of every nine deaths worldwide. Epidemiological studies conducted in this field have shown a solid connection between respiratory pathology and polycyclic aromatic hydrocarbon (PAH) exposure. COPD and asthma are respiratory diseases that were shown to have a strong association with exposure to PAHs. The purpose of the present study was to assess the serum levels of 15 PAHs in 102 COPD patients and to evaluate the results according to the residence environment of the investigated subjects. Analyses were carried out using a high-performance liquid chromatograph Nexera X2—Shimadzu Japan, which was equipped with an LC–30AD pump and an SIL–30AC autosampler. Spiked matrices, procedure blanks, spiked controls, and calibration standards in the acetonitrile were used as quality-assurance samples. Benzo(a)pyrene is the main representative of PAHs and was determined in higher concentrations in subjects with COPD versus the control group from the urban area (0.90/0.47 ng/mL) and rural area (0.73/0.44 ng/mL). The values obtained for the Benzo(a)pyrene-equivalent factor indicate a higher carcinogenic potential for patients diagnosed with COPD in urban areas compared to those in rural areas. These results could be due to traffic and vehicle emissions. This research identifies the need for legislative action to decrease semi-volatile organic compounds, especially PAHs, mainly in urban cities, in order to improve environmental management and health conditions.

## 1. Introduction

One of the leading risk factors for environmental health problems is air pollution. The World Health Organization (WHO) reports that this risk factor is associated with one of every nine deaths worldwide [1,2]. The term “air pollution” is usually attributed to a mixture of gaseous pollutants, like benzene, and other volatile organic compounds, PAHs, PCBs, PBDEs, pesticides, and metals [3]. The organic compounds formed from two or several condensed benzene rings are known as polycyclic aromatic hydrocarbons (PAHs). When organic materials suffer incomplete combustion, these organic compounds are formed, which is one of the main reasons why they are widespread pollutants [4]. Thus, a major contributors of PAHs in the atmosphere include diesel emissions, industrial installations, the burning of oil and coal as a source of energy, and residential heating [5]. Due to the development of the economic and industrial fields of the urban environment, high levels of pollutants were emitted. This caused an increased prevalence of chronic lung diseases [5,6]. Epidemiological studies conducted in this field have shown a solid connection between respiratory pathology and polycyclic aromatic hydrocarbon (PAH) exposure. The main respiratory diseases that were shown to have a strong association with exposure to PAHs are COPD and asthma, as well as respiratory infections and lung cancer [7,8,9]. The United States Environmental Protection Agency, or US EPA, acknowledge 16 PAHs as priority pollutants in the “Consent Decree”, of which seven are classified as potential human carcinogens: Indeno (1,2, 3-cd) pyrene, Benzo(a)pyrene (BaP), Chrysene (Chry), Benz(a)anthracene (BaA), Benzo(b)fluoranthene (BbF), and Dibenz(ah)anthracene (DahA) [10]. The mechanisms implicated in the induction or exacerbation of airway inflammatory responses by PAHs were studied over the years, while several are described in some review studies. When fine particles like PAHs are breathed into the lungs, they cause inflammation, which is one of the mechanisms implicated in the development, and also the exacerbation, of many respiratory diseases [11]. Research in the field of pollution is focused mostly on the urban environment [12] because it was found that most of the pollution is transmitted from outside to inside, while smoking and cooking contribute to internal pollution in a relatively stable interval [12]. Studies have also shown that more severe forms of COPD are seen in smokers and ex-smokers exposed to chemicals than in non-smokers. In addition, a lower level of systemic inflammation biomarkers was identified among non-smokers [11,13].

In Romania, data about the levels of PAHs in the blood samples of donors from urban and rural areas can be found because studies were conducted in this field [14], but an evaluation regarding the concentration levels of these pollutants in COPD patients has not yet been published. According to the reports of the ANPM in Romania, Iași is the second-most polluted city. In the last several years, the annual concentrations of PM_10_ did not exceed the considered normal values, but records of more than 35 daily exceedances during the year were registered, mostly in the city of Iași [15]. The purpose of this research was to assess the serum levels of 15 PAHs in 102 COPD patients and evaluate the results according to the residence environment of the investigated subjects. This is the first study that evaluates the level of PAH serum concentrations in COPD patients from the northeast area of Romania and attempts to highlight the main sources of exposure.

## 2. Materials and Methods

### 2.1. Characteristics of the Study Population

One hundred two patients participated in this research. The patients were hospitalized in the Clinical Hospital of Pulmonary Diseases, Iasi, Romania. Each participant signed an informed consent at the moment of inclusion. The 102 participants were divided based on the diagnosis of COPD, resulting in two groups: the COPD group and the control group. The control group was formed of patients diagnosed with other respiratory diseases. In Table 1, the clinical and demographic characteristics of the investigated patients can be found.

All biological samples were collected between September 2021 and February 2022. The samples were collected in the early hours of the morning in order to obtain 8 h of overnight fasting. Blood serum samples were preserved and stored at −25° Celsius. The biochemistry laboratory of the hospital was used for sample processing. Each participant had to answer a form that contained questions about area of residences, type of house heating, smoking habits (years of smoking, number of cigarettes smoked per day), and potential exposure through occupational and non-occupational factors. Information about age, gender, and comorbidities were also recorded.

### 2.2. Ethical Considerations

The study was approved by the ethics committee of the University of Medicine and Pharmacy “Grigore T. Popa” Iasi, Romania, and by the ethics committee of the Clinical Hospital of Pulmonary Diseases, Iasi, Romania (ethical approval no. 9205/9 June 2020, no. 83/22 April 2021, and no. 88/31 March 2022). Written informed consent for participation and for the use of medical data was obtained from all the participants in the study.

### 2.3. PAH Analyses

#### 2.3.1. Materials and Reagents

A standard mixture consisting of 16 PHAs, G1046205AL, DRE-L20950018AL, and internal standard Phenanthrene D.10 C 20920100—G20920100 was procured from Dr. Ehrenstorfer—LGC Standards (USA) and used for quantification. The qualitative composition and quantitative concentrations of PAHs were according to certificate of analysis, and their purities were of no less than 99.99%. Chromatographic method required methanol (MeOH), acetonitrile (ACN), dichloromethane (DCM), ethyl acetate (ETA) and water, which were all of HPLC purity, and were purchased from Merck KGaA in Germany. C18 cartridges Mega Bond Elute Plexa from Agilent Technologies USA (500 mg column bed, 6 mL volume) were used for sample preparation using solid-phase extraction on Supelco Visiprep SPE vacuum system.

#### 2.3.2. Instruments

Identification and quantification of PAHs were performed on Nexera X2 ultra-pressure liquid chromatograph from Shimadzu Japan. Standard configuration consisted of LC–30AD pump, SIL–30AC autosampler, CTO–30A column compartment, RF–10AXL FLD detector, and Lab Solution software. Pinnacle DB PAH Restek USA column with length, internal diameter, and particle size of 100 mm, 2.1 mm, and 1.9 μm was used.

#### 2.3.3. Chromatographic Method

Method parameters were established according to standard chromatographic practices, as in other studies [7,14], but with minor modifications. To separate PAH mixture components, a bicomponent mobile phase was used and consisted of water MF A and acetonitrile MF B. Method performance required gradient chromatographic elution of the two components of mobile phase at a constant flowrate of 0.54 mL/min. Starting mobile phase composition consisted of 50% acetonitrile MF B and 50% water MF A and was maintained for 8 min, then the acetonitrile MF B was gradually increased to 90% during 6 min and maintained at this level until Minute 18.5 (Table 2). Final equilibration required 1.5 min.

Column temperature was 30 °C. Chemical molecules of PAHs permitted detection on fluorescence detector and used wavelengths of excitation and emission (nm). Every category of PAHs had specific detection conditions that were also confirmed by previously mentioned study.

Due to the specificity of every component, wavelength optimization for excitation and spectral emission were performed according to the structure of analyzed samples. In addition, variations of gain amplitude were optimized in order to maximize the detection intervals.

#### 2.3.4. Standard Sample Preparation

Two consecutive stock solutions were prepared of 1250 ng/mL (SS1) and 125 ng/mL (SS2) from the main stock solution. For the preparation of standard solutions used in calibration, specified volumes of the two stock solutions were further used to produce a series of concentrations of 100 ng/mL (0.02 mL SS1), 75 ng/mL (0.15 mL SS2), 50 ng/mL (0.1 mL SS2), 25 ng/mL (0.05 mL SS2), 10 ng/mL (0.02 mL SS2), and 5 ng/mL (0.01 mL SS2) of each component.

Internal standard was Phenanthrene D10 (P.D10) prepared by dissolution of a quantity of 10 mg P.D10 in a volume of 100 mL acetonitrile. Next, a volume of 0.25 mL of previously prepared solution diluted to 100 mL using same solvent; the final concentration was 250 ng/mL.

#### 2.3.5. Sample Preparation

Biological samples were processed using 1-mL serum (plasma). A volume of 0.1 mL of internal standard stock solution (250 ng/mL) was added to each sample. To reduce viscosity, a dilution using 20 mL of water was performed. SPE cartridges were activated with 6 mL of DCM, 6 mL of MeOH, and 6 mL of H_2_O, followed by sample loading. To eliminate interreferences, column washing with 12 mL of H_2_O was included, followed by column drying for 30 min at high vacuum. Transfer of PAHs was performed by passing through SPE column a volume of 5 mL of DCM, which was evaporated under nitrogen to dry. The residue was reconstituted with 0.25 mL of can, and 5 μL was injected in the column.

#### 2.3.6. Method Validation

Validation was necessary because of the nature of the samples that were tested (biological fluids), but also because of the modifications required to assure the stability and reliability of the chromatographic method. Method validation consisted of testing the accuracy, precision with repeatability and intermediate evaluation, linearity, specificity, limits of detection, and limits of quantification in case of every quantified substance [16].

For accuracy, standard and spiked samples were used to verify the matrix effect or variations determined by the sample preparation procedures. Accuracy was evaluated on three spike levels established within the linearity range. Spike solutions were 100 ng/mL, 50 ng/mL, and 25 ng/mL. For spiking, a series of volumes from stock solutions of 0.02 mL of SS1, 0.1 mL of SS2, and 0.05 mL of SS2 were prepared using 1 mL of human serum. Before any other sample-preparation procedure, a volume of 0.1 mL of internal standard was introduced.

Linearity and range were considered according to method requirements of sensibility and limits of quantification. As mentioned, a 6 series of concentrations between 100 ng/mL and 5 ng/mL was included. Specificity was monitored using blank samples, standard solutions, and spike solutions, while interreference was evaluated from indigenous compounds that may rise from the biological samples. Precision and repeatability were evaluated for determination of recovery of the whole range of linearity using the three spiked samples of 100 ng/mL, 50 ng/mL, and 25 ng/mL. In addition, on the 50 ng/mL, a repeatability of determinations was evaluated for method relative standard deviations evaluation of parameters as retention time and area. Intermediate precision included further applications of the method in the same conditions by different analysts. Limits of detection and limits of quantifications were established according to the signal-to-noise ratio for the compounds at the lowest concentrations included in the study, but also using the statistical approach by standard errors of slopes.

#### 2.3.7. Method Development

**Figure 1:**
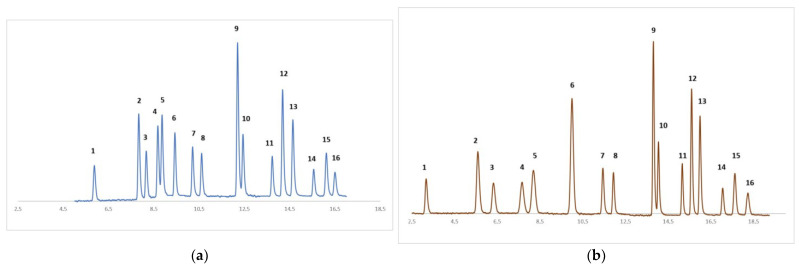
Method development. (**a**) 60% H_2_O:40% ACN (0.0–2.4 min), 10% H_2_O:90% ACN (2.4–14.0 min), 10% H_2_O:90% ACN (14.0–16.0 min), 60% H_2_O:40% ACN (16.0–16.25 min), flowrate 0.5 mL/min, temperature, 30 °C; (**b**) 50% H_2_O:50%ACN (0.0–8.0 min), 10% H_2_O:90% ACN (8.0–14.0 min), 10% H_2_O:90% ACN (14.0–18.5 min), 50% H_2_O:50% ACN (18.5–19.0 min), 50% H_2_O:50% ACN (19.0–21.0 min); flowrate 0.5 mL/min, temperature, 30 °C. Elution order: 1-N, 2-Ace, 3-Fl, 4-P, 5-P.D10, 6-A, 7-Flu, 8-Py, 9-BaA, 10-Ch, 11-B(b)Flu, 12-B(k)Flu, 13-BaPy, 14-D(ah)A 15-BghiPY, 16-IPy.

Method development was initiated with selectivity of all 16 target compounds. In the literature, there are many approaches for determination of PAHs on special dedicated columns, such as PAHs columns, which are C18 analytical columns but possess specific carbon load [17,18]. Specificity of compounds of fused aromatic rings [19] requires mobile phases that usually use mixtures of water and acetonitrile. In this case, the separation mechanism is polarity affinity because the fused aromatic rings of the PAHs do not indicate any ionization [18]. Some problems regarding the separation of certain compounds were revealed [20]. Most important chromatographic challenge was determined by separation between internal standard P.D10 and P. Several approaches were tested, but the best results were for chromatographic method that required an isocratic section for first 8 min, then the gradient was involved for further 10.5 min (Figure 1b). First, a faster gradient was applied with the same final conditions (10% H_2_O:90% can), but there were some problems regarding separation of P and P.D10 but also B(b)Flu and B(k)Flu (Figure 1a). Using a final composition of mobile phase of 90% acetonitrile:10% water permitted the separation of the two compounds and assured further separation of D(ah)A, BghiPY, and IPy on 16.5 min. Two minutes were further necessary to re-equilibrate the column to the starting conditions of the mobile phase, as presented in Figure 1.

Second step was wavelength selection of excitation and emission wavelength in order to improve method sensitivity. Fluorescence parameters were considered from other studies but were assessed according to elution-retention times of the determined compounds. As a result of this selection, some of the compounds showed a weak signal, while others presented signal that was out of linearity range of the detector. The values used are in a wide range of wavelengths for excitation and emissions; they are strongly dependent of fluorescence detector and of the conditions provided by the mobile phase [19,21].

For the best optimization of excitation and emission wavelengths used in FLD, some intervals were set around 270 ± 20 nm for excitation and 400 ± 50 nm for emissions [22]. In addition, the gain amplifications were optimized in function of signal intensity. The correlation between excitation, emission wavelengths, and the gain amplification were performed in function of the number of aromatic rings in the molecule. In this correlation, first five compounds, such as N, Ace, Fl, P, and P. D10, were identified by setting values of excitation of 270 ± 5 nm and emissions of 350 nm, less than 400 nm. For the following series, with four aromatic rings (Flu, Py, BaA, Ch), detection was performed around same excitation wavelength but with higher values for emission 430 ± 10 nm. Finally, the series of compounds with a higher number of fused aromatic rings were evaluated at 290 nm for excitation and 470 nm of emission. Special situation was considered for BaA, Ch, Per, B(b)Flu B(k)Flu, and BaPy, which required identical excitation (260 nm) and emission (420 nm) wavelengths but different gain amplitudes.

Sample preparation was the final step in method development. Use of solid-phase extraction is widely used for sample clean-up and enrichment [23]. Several methods for PAHs were applied, but these methods were developed for environmental samples of water, soil, and other sample types [24]. PAHs in biological tissues as human plasma are present because of the important sources of provenience as smoking and occupational exposure [25]. These compounds are partially metabolized, and the presence in these biological media is not affected by protein binding [26]. Therefore, the compounds are present in free form, so the determination from these samples is possible as in other types of samples by using solid–liquid extraction using deproteinization, solid-phase extraction, or liquid–liquid extraction [27]. Limits of determination found in several studies employed the use of solid-phase extraction, usually with C18 cartridges.

For this approach, activation of cartridges with MeOH, DCM, and ETA in several proportions and different mixtures of wash solutions were tested. Column clean-up using solvent with low eluotropic strength, such as H_2_O and H_2_O with several proportions of organic solvents, was included in the study. Finally, the elution was conducted by testing more organic solvent mixtures in order to maximize the recovery on column.

Conditioning with DCM:ETA (50:50%) (2 mL), MeOH (2 mL) followed by washing with water (2 mL), and elution with same mixture DCM:ETA (50:50%) (2 mL) was tested. Sample was 1 mL of human plasma. In this situation, because of the viscosity of the sample, some of the compounds with higher lipophilicity were lost during sample loading, the cleanup was incomplete, and then elution recovery was below 50% for compounds such as BaA, Ch, Per, B(b)Flu B(k)Flu, BaPy, and D(ah)A BghiPY Ipy (See Figure S1a in Supplementary Materials). To improve the recovery, some modifications were performed by using activation procedure with DCM (2 mL), MeOH (2mL), and H_2_O (2 mL). The 1-mL volume of sample was diluted to 10 mL with a solution of 20% MeOH:80%H_2_O. The elution remained the same, but in these conditions, the compounds with lower aromatic rings and higher polarity (N, Ace, Fl, P, P.D10, A) were better retained on the column. However, the recovery comprised between 20% for N and 45% for P. The other compounds with higher affinity had a better recovery of Flu, Py, BaA, and Ch, with recoveries between 56–98% (See Figure S1b in Supplementary Materials). In the final step of the method, activation was conducted by using a higher volume (6 mL) for DCM, MeOH, and water, while the volume of 1 mL of plasma was diluted to 20 mL with H_2_O. In addition, the volume of washing was increased to 12 mL of H_2_O, and the elution was conducted with 5 mL of DCM. Complete elution was confirmed by the use of a second elution volume of DCM, while the use of higher volume of water for wash did not affect the interactions of PAHs with stationary phase, and the cleanup was superior. The latest three modifications are presented in Figure S1c in Supplementary Materials.

#### 2.3.8. Method Validation

##### Specificity

**Figure 2:**
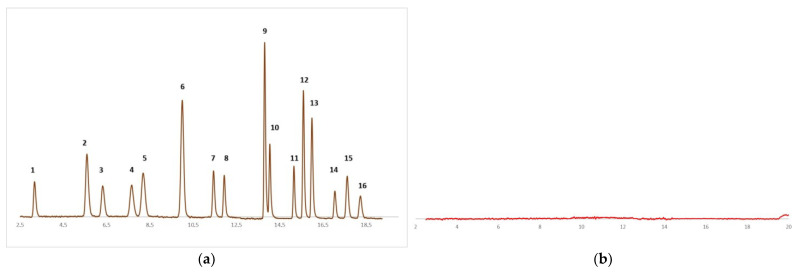
(**a**) Standard solution (**b**) Blank solution.

Specificity was assessed using standard solutions, blank samples, and spiked samples. Specificity followed the evaluation of interference degree from the indigenous compounds that could be present from the human plasma. From the following chromatograms, there is no interference from the sample preparation. No peak was present except on the chromatogram. Representative chromatograms are presented in Figure 2.

Elution order: 1-N, 2-Ace, 3-Fl, 4-P, 5-P.D10, 6-A, 7-Flu, 8-Py, 9-BaA, 10-Ch, 11-B(b)Flu, 12-B(k)Flu, 13-BaPy, 14-D(ah)A 15-BghiPY, 16-IPy.

##### Linearity

Linearity was established by preparing several solutions with concentrations between 100 ng/mL and 5 ng/mL. The level of 100 ng/mL was upper limit of quantification and was established as the first concentration from which the chromatographic peaks fulfilled the conditions of system suitability with the asymmetry between 0.8 to 1.5, and the theoretical plates were of minimum 20,000. Using higher concentrations, the asymmetry of chromatographic peaks did not fulfill the required conditions, while the peaks for Flu, Py, Per, B(b)Flu B(k)Flu, and BaPy oversaturated the detector.

In this linearity range, the samples were analyzed in triplicate. Relevant parameters were slope, correlation coefficient, standard error of slope (Sy), and statistical evaluation (p,T) for regression.

Analysis of variance for regression parameters showed a significant correlation between peak areas and concentrations of PAHs. Data showed that the necessary correlation conditions were satisfied to produce the evaluation of concentrations using peak area. Previously mentioned conditions were verified using slope *p*-value that had values less than 0.05 (Table S1 Supplementary Materials).

##### Limits of Detection and Limits of Quantification

Limits of detection (LOD) and quantification (LOQ) revealed the lowest concentration of the PAHs (LOD) and quantified with sufficient accuracy (LOQ). These limits were statistically confirmed by recordings for blanks prepared using sample procedure. No interferences in baseline amplitude were found that could affect the LOQ and LOD for each PAH. In blank chromatograms, no peaks with similar retention times with PAHs were not recorded or were close to signal-to-noise ratio. In evaluation of LOD and LOQ, response factor was used and calculated as ratio of analyte quantity and the height of the corresponding peak. LODs and LOQs were also estimated using standard errors (Sy) and slope values (Table 3). Values for LODs comprised between 0.003 ng/mL for A and 0.45 ng/mL for Ch. The range of concentrations for LOQ were between 0.01 and 1.35 ng/mL. These levels are in accordance with other findings [19].

##### Repeatability and Intermediate Precision

Precision was defined through repeatability and intermediate precision, which comprised applying the method conditions with all the steps in several conditions determined by applying the method by two analysts and two instruments. Applying the method in these conditions, the parameters, such as retention time and estimated concentration based on calibration curves, for every compound were tested. The average values are presented in the Table S2 Supplementary Materials.

##### Accuracy and Matrix Effect

In sample preparation method development, clean-up step was used to eliminate interfering matrix components because of the lack of specificity of FLD-detection method. For this procedure, C18 cartridges were used with 600-mg bed to assure capacity of retaining the compounds.

Higher retaining capacity of octadecylsilane stationary phase also assured elimination of lipids and proteins from biological samples. Results for this procedure are presented in Table 4. To establish recovery factors for every PAH, spiked samples with 100, 50, and 250 ng/mL of every compound were prepared. Every concentration level was reproduced in triplicate. Recovery factors on every compound were calculated based on the individual Slope C.

Distribution of concentrations found ranged between 90.5% and 112.3% for the concentration of 100 ng/mL and 83% and 132.6% for the lower level of 25 ng/mL. In addition, the relative standard deviation comprised between 0.69% Ipy and 1.53% for A in case of C1 and 2.05% D(ah)A and 3.83% for Flu in case of C2. A particular situation was 25 ng/mL concentration, for which RSD was between 2.78% for P and 6.29% for BaPy. At lower concentrations that are close to the LOQ, considering the high number of steps for sample preparation, the relative standard deviation is near 5%, which assured good precision and accuracy.

##### Statistical Analysis

The software package STATISTICA 10 (StatSoft Inc. Oklahoma, United States) was used to perform the statistical analyses. Kolmogorov–Smirnov test was used to assess the normal distribution. Spearman’s rank correlation coefficient was used to measure the strength of the correlations. A *p*-value < 0.05 was considered indicative of statistical significance. The characteristics of the two groups divided according to area of residence were compared using the Mann–Whitney U test. This test was also used to assess if the two groups of patients differ regarding the PAH concentrations. For the identification of a possible source of exposure, factorial analysis was performed. The Microsoft Excel program was used to perform the descriptive statistic in case of urban/rural areas and, for each PAH compound, determine the patient’s average, standard deviation, median, and range.

## 3. Results

### Concentration Profile of PAHs in Blood Serum in Urban/Rural Donors

In each of the two groups, COPD group and control group, 51 patients were included. The second group consisted of patients diagnosed with pulmonary tuberculosis (n = 15), pneumonia (n = 7), and other respiratory diseases. A statistical assessment of the clinical characteristics and the biological parameters was performed. Table 1 shows the demographic characteristics of the patients that were included in this research. The median age of the COPD group was 59.48 years, as well as 55.01 for control group. The male gender was the most predominant in the whole lot of patients. In the COPD group, 57% are former smokers, compared to 6% in the control group; only 18% of COPD group are non-smokers, against 57% in the control group, while 16% of COPD group is professionally exposed to PAHs against 1% in the control group. Most of the patients with COPD were diagnosed with Stages II (24%), III (43%), and IV (29%). Regarding comorbidities, hypertension was predominant in the COPD group (49%), as well as in the control group (24%). Diabetes (14%) and obesity (14%) were prevalent in the COPD group.

In the present study, blood serum-concentration levels for 15 priority PAHs were determined.

The Mann–Whitney U test was used to statistically analyze the data recorded after the separation of the main lot into four groups: rural control, urban control, rural COPD, and urban COPD. The results obtained in urban and rural patients showed that PAH concentrations (BaA, B(b)Flu, D(ah)A, IPy, BghiPy, N, Ace, Fl, A, Flu, and Py) differ significantly (*p* ˂ 0.0001) for the groups Urban COPD × Urban Control, Rural COPD × Urban Control, Rural Control × Urban COPD. In the case of Rural Control × Urban Control, these groups differed only by BghiPy (*p* = 0.044). The most abundant carcinogenic PAHs were D(ah)A and were quantified in the urban COPD group. The potency of DahA was assessed to be 10 times greater than the potency of BaPy [28]. In order to assess the relationships between the investigated parameters and the main sources of exposure, factorial analysis was applied. Factorial analysis is a statistical tool with wide applicability regarding the identification of exposure sources for both environmental and biological matrices.

The mean, median, standard deviation, and range for the blood concentrations of PAHs (ng/mL) in the main two groups (COPD and control) are shown in Table 5 and Table 6.

## 4. Discussion

The profile of PAH concentrations is represented by higher concentrations of non-carcinogenic PAH compounds both for the control group (with an arithmetic mean in the urban area, ΣPAHs = 14.63 ± 13.97 ng/mL and ranged from 1.30–65.25 ng/mL, while for the rural area, ΣPAHs = 12.59 ± 11.15 ng/mL and ranged from 1.46–43.71 ng/mL), as well as for the COPD group (in the urban area, ΣPAHs = 12.91 ± 17.72 ng/mL and ranged from 1.92–74.85 ng/mL, while in the rural area, ΣPAHs = 15.61 ± 17.35 ng/mL and ranged from 2.73–71.98 ng/mL).

Regarding the lower concentrations of PAHs in urban COPD patients, several hypotheses can be formulated considering studies carried out in the field during the pandemic since the samples were collected between September 2021 and February 2022. One of the hypotheses that was considered is related to the fact that there were numerous restrictions during the pandemic (traffic, the obligation to wear a protective mask in open spaces, etc.) [29]. The second hypothesis is related to the fact that studies conducted in this period of time also showed that patients with different types of comorbidities isolated themselves, which can be related to lower exposure to outdoor pollutants [30]. The fact that higher concentrations of PAHs were found in COPD patients from rural areas can be explained considering that samples were collected during the cold season and patients were exposed to indoor pollution caused by domestic heating. However, a higher concentration of PAHS can be observed in the control group. This result can be explained by higher exposure to other sources of PAHs, like smoking (control group comprised 37% smokers, while COPD group comprised 25% smokers) (Table 1). Comparative studies of PAHs during the pandemic period and PAHs during the non-pandemic period revealed a gradual reduction in PAH concentrations in the atmosphere due to the restrictions imposed in some areas of activities such as construction, industries, transportation, and traffic roads [30].

**Figure 3:**
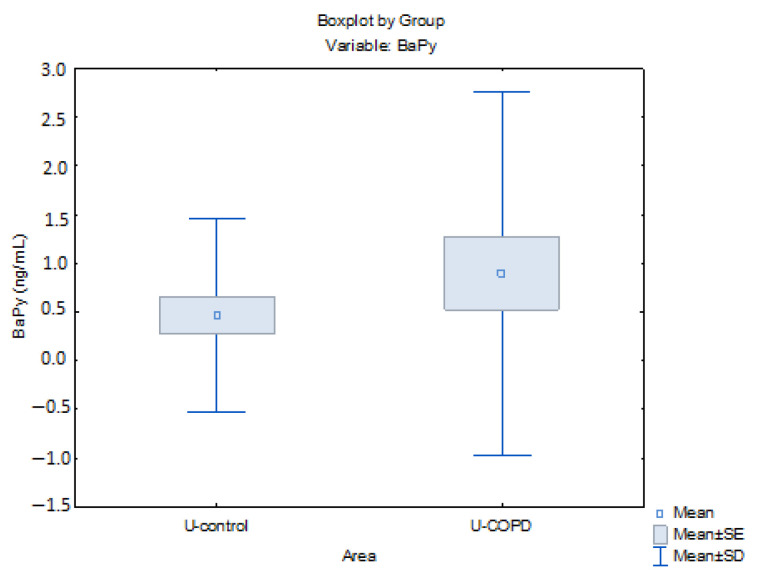
Level of BaPy (ng/mL) in Urban–control/Urban–COPD.

Naphthalene and phenanthrene were the most abundant non-carcinogenic PAHs in both groups. Moreover, in the control group, we observed high concentrations of non-cancerous PAHs in the blood, especially naphthalene, with mean concentration values of 9.63 ng/mL and 8.73 ng/mL for urban and rural environments. Since naphthalene is released more frequently, especially in motor vehicle exhaust, this can explain its higher contribution to the total levels of PAHs in urban areas [31]. Phenanthrene was determined within the same concentration limits for both urban and rural areas (COPD group urban/rural area = 2.62/2.65 ng/mL), as well as the control group (urban/rural area = 2.64/2.51 ng/mL). Concentrations for Py, Ch, and BghiPy were elevated in serum samples from the urban COPD group compared to values associated with the urban control group (Py: 1.57 ng/mL vs. 0.31 ng/mL; Ch: 1.07 ng/mL vs 0.90 ng/mL; BghiPy: 1.27 ng/mL vs. 0.35 ng/mL). Recent studies have shown that the levels of phenanthrene and pyrene are particles related to the emission gases produced by diesel fuels but can also result from the burning of fossil fuels and biomass [32]. This is why Phenanthrene, Pyrene, and Benz (a)Pyrene are considered by Sobus el al. (2008) biomarkers of exposure [33]. In another study published in 2009, Sobus et al. concluded that naphthalene and phenanthrene urine levels reflect atmospheric exposure to the compounds that, in the future, can be used as surrogates for occupational exposure to PAHs [34]. Reactive electrophilic metabolites and the activation of cellular receptors are hypothesized to be related to PAH toxicity, with one of these receptors being the aryl hydrocarbon receptor (AhR) [35,36,37]. BaPy is the main representative of PAHs and was determined in higher concentrations in subjects with COPD versus the control group from the urban area (0.90/0.47 ng/mL) and rural area (0.73/0.44 ng/mL) ( Table 5 and Table 6). These results for patients from urban areas are presented in Figure 3. The Kolmogorov–Smirnov test, applied by a variable area of residence in the Urban Control–Urban COPD groups, presented a significant correlation (*p* < 0.005). Epidemiological studies have shown that BaPy activates AhR, a transcription factor contained in all tissues, including bronchial epithelial cells, and also has a major role in the development of COPD and lung cancer [38].

Regarding the profile of carcinogenic PAHs, represented by compounds with 4–6 benzene rings, the results of the descriptive statistics show differences for the two investigated groups.

Thus, the concentration profile of carcinogenic PAHs corresponding to the group of COPD patients from the urban environment were significantly higher (ΣPAHs = 5.78 ng/mL) compared to the concentration values for the urban patients corresponding to the control group (ΣPAHs = 2.05 ng/mL). The results of studies published in this field show that the benzene-soluble fraction that is formed by PAHs with 4–7 benzene rings from vehicle exhaust, coal stove emissions, and tobacco smoke is directly related to the carcinogenic risk imposed by PAHs that resulted from these types of sources [39]. Regarding tobacco smoke exposure, the statistical results obtained using the Mann–Whitney U test comparing non-smokers and former smokers from the urban area reveal significant differences for the following carcinogenic PAHs: B(b)Flu *p* = 0.01, D(ah)A *p* = 0.02, and IPy *p* = 0.03. In addition, between non-smokers and smokers from the urban area, significant differences for the following carcinogenic PAHs are revealed: IPy *p* = 0.04. It was observed that the most noticeable carcinogenic effect of tobacco smoke (100 ng BaPy/per cigarette) on the lungs was caused by the fraction of four or more benzene rings [39].

**Figure 4:**
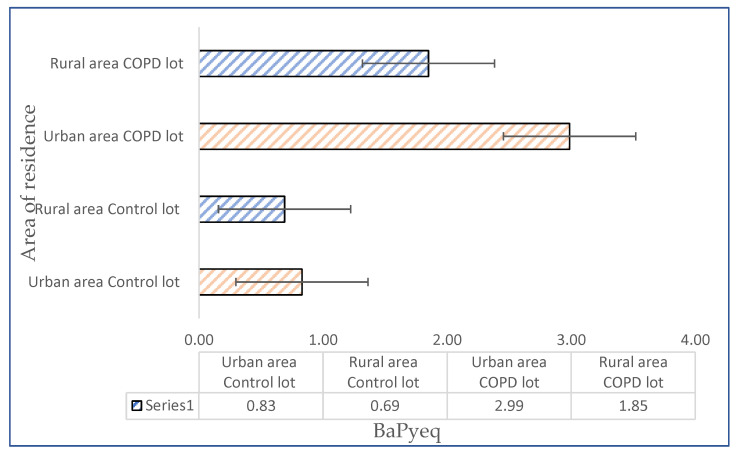
BaPyeq determined in blood serum samples for COPD and control group patients residing in urban and rural areas.

In the present study, the benzo–pyrene–equivalent factor (BaPyeq) was calculated in an attempt to determine the carcinogenic potencies of the serum levels of PAH. The calculation was carried out by multiplying the concentrations of each PAH by its toxic equivalence factor for cancer potency in relation to BaPy. According to the USEPA, the calculated TEFs for BaA is 0.1; for BaPy, it is 1; for BbFlu, it is 0.1; for BkFlu, it is 0.001; for Ipy, it is 0.1; for DahA, it is 1; and for Ch, it is 0.001 [7]. The values obtained for BaPyeq indicate a higher carcinogenic potential for COPD patients from urban areas compared to COPD patients that reside in rural areas (Figure 4).

PAHs resulted from traffic and motor vehicle emissions explain the results that show a difference in blood samples from patients in the two investigated areas. The results obtained in the blood samples cannot be connected to a specific origin even if environmental exposure would be a fairly stable cause. Otherwise, data from the literature have shown that 60% of PAH concentrations in the air result from vehicle emissions [40,41].

The statistical difference generated with the Mann–Whitney U test for Rural Control × Urban Control could explain diesel exposure to a large extent because 57% of the patients included in the control group are non-smokers (Table 1). Recent studies carried out in Romania showed different concentrations of PAHs in serum for donors from the urban and rural areas, especially for the N, Fl, P, and Py [14]. Many epidemiological studies involving a large number of participants have observed that compounds with 2–4 benzenic rings, such as N, P, Fl, and Py, as well as compounds with more than four benzenic rings, were associated with obstructive lung diseases [42].

In this study, the factorial analysis was performed for COPD patients and from the control group according to the area of residence. Thus, the results of the factorial analysis scored three factors (Table 7) that significantly contribute to the separation of the data for the control group and COPD group.

**Figure 5:**
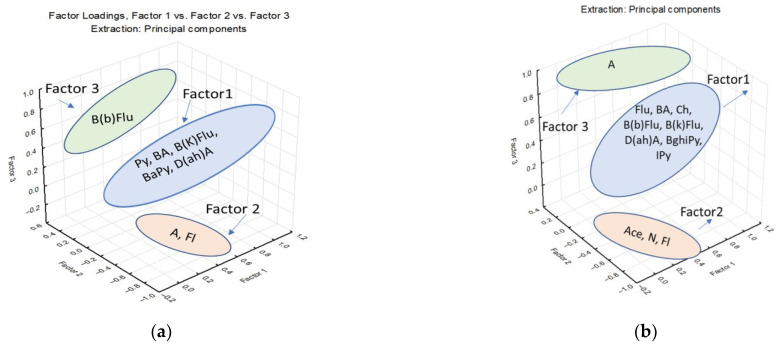

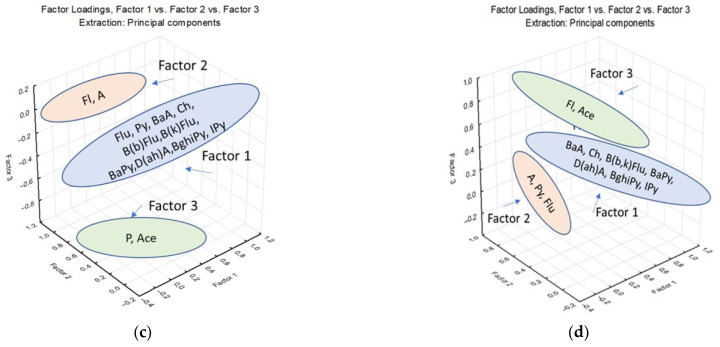
(**a**) Factor lodgings in control group rural area; (**b**) Factor lodgings in COPD group rural area; (**c**) Factor lodgings in control group urban area; (**d**) Factor lodgings in COPD group urban area.

The contribution of the first factor is represented by a preponderance of compounds with 4–6 benzene rings in all investigated groups (Figure 5).

Comparing rural and urban areas for the COPD group, we observe a contribution of the first factor of 57.28%/53.67% of the total variation of the dataset. The abundance of PAHs in rural serum samples could be due to the fact that most people heat themselves in the cold season with wood-based stoves; these emissions represent an important source of PAHs. Factor 2 and Factor 3 are found in a low contribution in the total variation of the dataset and are mostly represented by non-carcinogenic PAHs [28].

## 5. Conclusions

The present study evaluated 15 EPA (USA)-priority PAHs in serum samples of COPD patients and in serum samples of patients diagnosed with other lung diseases. Analyses were carried out using a high-performance liquid chromatograph Nexera X2—Shimadzu Japan, equipped with a LC–30AD pump, SIL–30AC autosampler. Spiked matrices, spiked controls, procedure blanks, and calibration standards in acetonitrile were used as quality-assurance samples. The method’s limits of quantification (LOQ) for individual PAHs were between 0.01 and 1.35 ng/mL. Benzo(a)pyrene is the main representative of PAHs and was determined in higher concentrations in subjects with COPD versus control from the urban area (0.90/0.47 ng/mL) and the rural area (0.73/0.44 ng/mL). The Kolmogorov–Smirnov test, applied by the variable area of residence in the Urban Control–Urban COPD groups, presented a significant correlation (*p* < 0.005). Naphthalene and phenanthrene were the main contributors to the final results of non-carcinogenic PAHs. The concentration profiles of the 15 PAHs are represented by high concentrations of non-carcinogenic compounds both for the control group (urban area ΣPAHs = 14.63 ng/mL, rural area ΣPAHs = 12.59 ng/mL), but also for the COPD group (urban area ΣPAHs = 12.91 ng/mL, rural area ΣPAHs = 15.61 ng/mL). Considering the period of the sample collection (September 2021–February 2022), the abundance of compounds in rural serum samples could be due to the fact that most people heat themselves in the cold season with wood-based stoves, as these emissions represent an important source of PAHs. The values obtained for BaPyeq indicate a higher carcinogenic potential for patients diagnosed with COPD in urban areas compared to those in rural areas. These results could be due to traffic and vehicle emissions. This research identifies the need for legislative action to decrease semi-volatile organic compounds, especially polycyclic aromatic hydrocarbons, mainly in urban cities, as well as the need to improve environmental management and health conditions. Moreover, extensive research is needed, which includes a larger number of biological matrices to identify the main sources of exposure.

## Figures and Tables

**Table 1:** Clinical and demographic characteristics.

Patient Characteristics	COPD Group (n = 51)	Control Group (n = 51)
Variables		
Age (years)	59.48 ± 10.96	55.01 ± 14.39
Height (cm)	171 ± 0.05	168.6 ± 0.07
Weight (kg)	67.2 ± 8.92	65.3 ± 9.67
Gender		
Male	47 (92%)	32 (63%)
Female	4 (8%)	19 (37%)
Area of residence		
Rural	25 (49%)	24 (47%)
Urban	26 (51%)	27 (53%)
Smoking status		
Former smokers	29 (57%)	3 (6%)
Non-smokers	9 (18%)	29 (57%)
Smokers	13 (25%)	19 (37%)
Packs-years (PY)	34 ± 19.97	22.61 ± 10.68
Primary diagnostic	COPD	Tuberculosis 15 (29%)
COPD stage I	2 (4%)	Bronchial asthma 4 (8%)
COPD stage II	12(24%)	Bronchitis 3 (6%)
COPD stage III	22(43%)	Pneumonia 7 (14%)
COPD stage IV	15(29%)	Other diseases 22 (43%)
Profesional exposure to PAHs	16%	1%
Passive smoking	10%	8%
Comorbidities		
Hypertension	25 (49%)	12 (24%)
Diabetes mellitues	7 (14%)	2 (4%)
Obesity	7 (14%)	3 (6%)

**Table 2:** Chromatographic method for PAHs separation.

Time (min)	MF A (H_2_O)	MF B (ACN)	Elution
0–8.0	50	50	Isocratic
8.0–14.0	10	90	Gradient
14.0–18.5	10	90	Isocratic
18.5–19.0	50	50	Gradient
19.0–21.0	50	50	Isocratic—equilibration

**Table 3:** Excitation and emission wavelength for the compounds in the study.

Time (min)	Excitațion (nm)	Emission (nm)	Gain	Category of Compounds
0–9.1	275	350	16×	N, Ace, Fl, P, P.D10
9.1–9.7	260	420	16×	A
9.7–11.5	270	440	16×	Flu, Py
11.5–13.5	260	420	16×	BaA, Ch
13.5–15.0	260	420	4×	B(b)Flu B(k)Flu, BaPy
15.0–17.0	290	470	16×	D(ah)A BghiPY IPy

**Table 4:** Evaluation of recovery factors, standard error, and relative standard deviations on three series of samples prepared using proposed procedure at three levels of concentrations.

PAHs	C1 (100 ng/mL)			C2 (50 ng/mL)			C3 (25 ng/mL)		
	Mean Rec (%)	S.D. (n = 3)	RSD (%)	Mean Rec (%)	S.D. (n = 3)	RSD (%)	Mean Rec (%)	S.D. (n = 3)	RSD (%)
**N**	103.5	1.25	1.21	108.5	1.23	2.27	106.8	1.01	3.78
**Ace**	100.7	1.2	1.19	103.2	1.89	3.66	93.4	0.98	4.20
**Fl**	105.5	1.1	1.04	101.4	1.67	3.29	100.4	1.02	4.07
**P**	101.7	1.06	1.04	110.0	1.86	3.38	132.6	0.92	2.78
**A**	98.0	1.5	1.53	98.4	1.59	3.23	83.0	0.86	4.14
**Flu**	99.6	1.45	1.46	92.5	1.77	3.83	105.4	0.92	3.49
**Py**	93.0	1.28	1.38	103.2	1.52	2.95	105.6	0.97	3.67
**BaA**	90.5	1.27	1.40	109.5	1.28	2.34	108.5	0.86	3.17
**Ch**	103.7	1.3	1.25	110.6	1.37	2.48	95.0	0.91	3.83
**B(b)Flu**	96.2	1.26	1.31	113.6	1.36	2.40	108.3	1.26	4.65
**B(k)Flu**	101.3	1.27	1.25	110.3	1.34	2.43	93.6	1.24	5.30
**BaPy**	92.3	1.23	1.33	110.9	1.33	2.40	85.3	1.34	6.29
**D(ah)A**	108.7	1.08	0.99	109.2	1.12	2.05	87.4	1.27	5.81
**BghiPY**	105.8	0.99	0.94	104.7	1.2	2.29	100.8	0.99	3.93
**IPy**	112.3	0.78	0.69	113.5	1.2	2.11	108.8	0.82	3.01

**Table 5:** Carcinogenic and non-carcinogenic PAHs concentrations (ng/mL) in human blood serum of patients selected for COPD group.

COPD Group	Urban Area				Rural Area			
	(n = 26)				(n = 25)			
Non-Carcinogenic PAHs	Mean	Median	Stdev	Range	Mean	Median	Stdev	Range
N	4.48	3.38	4.85	0.01–18.15	8.39	5.82	9.27	0.80–37.90
Ace	0.38	0.28	0.31	0.28–1.70	0.29	0.28	0.07	0.28–0.61
Fl	0.36	0.16	0.49	0.05–1.66	0.51	0.16	0.63	0.16–2.37
P	2.62	2.21	1.52	1.01–8.41	2.65	2.40	1.08	0.91–5.26
A	0.23	0.21	0.11	0.06–0.66	0.51	0.21	1.06	0.07–4.05
Flu	0.93	0.20	2.15	0.20–10.45	0.64	0.20	0.97	0.20–3.96
Py	1.57	0.26	2.77	0.01–11.19	0.97	0.26	1.15	0.26–4.02
Ch	1.07	0.21	2.28	0.02–10.18	0.92	0.25	1.63	0.04–6.96
BghiPY	1.27	0.29	3.24	0.29–16.04	0.72	0.29	1.48	0.01–6.83
**ΣPAHs**	**12.91**	**7.20**	**17.72**	**1.92–74.85**	**15.61**	**9.88**	**17.35**	**2.73–71.98**
**Carcinogenic PAHs**								
BaA	0.82	0.16	1.66	0.01–7.39	0.67	0.16	1.47	0.01–5.76
B(b)Flu	0.97	0.26	2.01	0.21–9.99	0.98	0.26	2.96	0.21–14.77
B(k)Flu	0.97	0.18	2.09	0.07–9.07	0.87	0.16	1.94	0.09–8.79
BaPy	0.90	0.22	1.87	0.09–7.38	0.73	0.28	1.19	0.09–4.20
D(ah)A	1.24	0.36	2.84	0.05–12.55	0.88	0.36	1.28	0.01–5.98
IPy	0.89	0.20	2.17	0.03–9.25	0.72	0.20	1.88	0.19–9.01
**ΣPAHs**	**5.78**	**1.38**	**12.63**	**0.46–55.63**	**4.86**	**1.42**	**10.72**	**0.60–48.51**

**Table 6:** Carcinogenic and non-carcinogenic PAH concentrations (ng/mL) in human blood serum of patients selected for control group.

Control Group	Urban Area				Rural Area			
	(n = 27)				(n = 24)			
Non-Carcinogenic PAHs	Mean	Median	Stdev	Range	Mean	Median	Stdev	Range
N	9.63	9.30	5.95	0.01–25.92	8.73	7.52	7.00	0.1–25.65
Ace	0.16	0.01	0.54	0.01–2.70	0.12	0.01	0.38	0.1–1.80
Fl	0.31	0.03	0.88	0.01–4.13	0.32	0.02	0.59	0.01–1.63
P	2.64	2.57	1.03	1.30–4.89	2.51	2.28	0.81	1.46–4.08
A	0.04	0.01	0.11	0.01–0.54	0.04	0.01	0.10	0.01–0.39
Flu	0.29	0.03	0.84	0.02–3.60	0.21	0.02	0.42	0.01–1.36
Py	0.31	0.02	0.82	0.02–3.80	0.29	0.01	0.91	0.01–4.17
Ch	0.90	0.40	2.74	0.01–14.51	0.35	0.06	0.83	0.01–4.07
BghiPY	0.35	0.01	1.07	0.01–5.16	0.02	0.01	0.11	0.01–0.54
**ΣPAHs**	**14.63**	**12.27**	**13.97**	**1.30–65.25**	**12.59**	**9.86**	**11.15**	**1.46–43.71**
**Carcinogenic PAHs**								
BaA	0.26	0.13	0.92	0.04–4.76	0.22	0.01	0.82	0.03–4.03
B(b)Flu	0.23	0.17	1.11	0.04–5.79	0.09	0.02	0.21	0.01–0.64
B(k)Flu	0.57	0.17	1.96	0.02–10.33	0.38	0.15	0.88	0.02–4.13
BaPy	0.47	0.24	1.00	0.08–4.12	0.44	0.30	0.86	0.06–4.18
D(ah)A	0.28	0.01	1.38	0.01–7.17	0.21	0.06	0.82	0.01–3.99
IPy	0.23	0.01	1.08	0.01–5.64	0.13	0.08	0.49	0.01–2.41
**ΣPAHs**	**2.05**	**0.31**	**7.45**	**0.20–37.81**	**1.48**	**0.45**	**4.08**	**0.14–19.38**

**Table 7:** Extraction of principal components for the control group and COPD group.

Extraction Principal Components	Factor 1 % Total Variance	Factor 2 % Total Variance	Factor 3 % Total Variance
COPD_urban area_	53.67	15.80	10.42
COPD_rural area_	57.28	14.39	12.75
CONTROL_rural area_	39.54	12.78	11.07
CONTROL_urban area_	60.38	12.96	8.51

## Data Availability

Not applicable.

## Supplementary Materials

The following supporting information can be downloaded at: https://www.mdpi.com/article/10.3390/jpm13091290/s1, Figure S1. Method development, SPE conditions. (a) DCM:ETA (50:50%) (2 mL), MeOH (2 mL), H_2_O (2 mL), load plasma + 100 ng/mL PAHs + 100 ng/mL P.D10 (1 mL), wash 2 mL H_2_O, elution DCM:ETA (50:50%) (2 mL); (b) DCM (2 mL), MeOH (2 mL), H_2_O (2 mL), load plasma + 100 ng/mL PAHs + 100 ng/mL P.D10 (1 mL) + 9 mL MeOH:H_2_O (10:90%), wash 2 mL H_2_O, elution DCM:ETA (50:50%) (2 mL); (c) DCM (6 mL), MeOH (6 mL), H_2_O (6 mL)), load plasma + 100 ng/mL PAHs + 100 ng/mL P.D10 (1 mL) + 19 mL MeOH:H_2_O (10:90%), wash 12 mL H_2_O, elution DCM:ETA (50:50%) (5 mL). Elution order: 1-N, 2-Ace, 3-Fl, 4-P, 5-P.D10, 6-A, 7-Flu, 8-Py, 9-BaA, 10-Ch, 11-B(b)Flu, 12-B(k)Flu, 13-BaPy, 14-D(ah)A 15-BghiPY, 16-IPy; Table S1. Linearity parameters; Table S2. Stability of retention time and estimated concentrations using regression slopes.
