# Supplementary material for: HPLC Analysis and Risk Assessment of 15 Priority PAHs in Human Blood Serum of COPD Patient from Urban and Rural Areas, Iasi (Romania)
Source: J Pers Med. 2023 Aug 23;13(9):1290. doi: 10.3390/jpm13091290 (PMC10532781; doi:10.3390/jpm13091290)
Supplement: Supplementary file 1 [file jpm-13-01290-s001.zip › jpm-2520496-supplementary.pdf]

Supplementary Materials

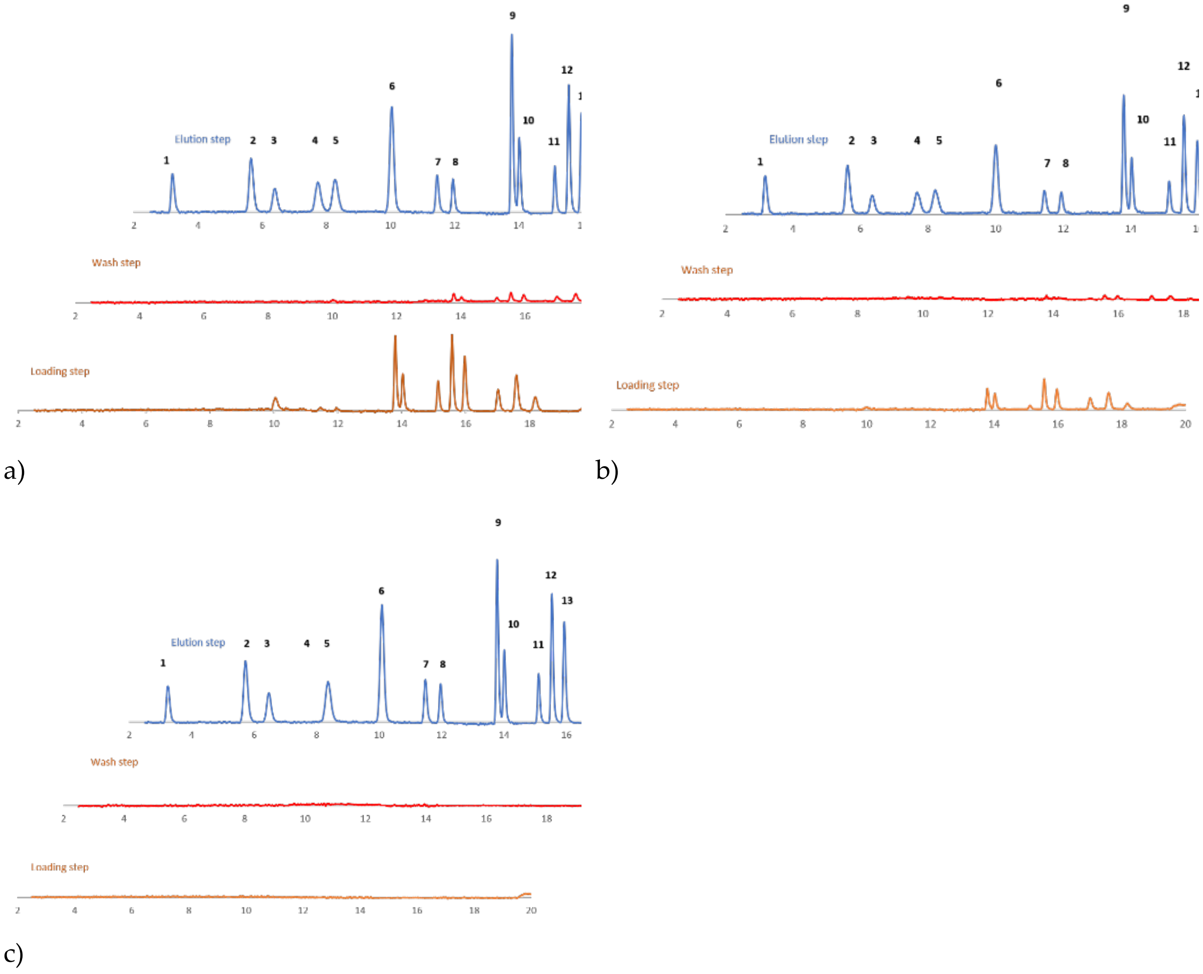

Figure S1. Method development, SPE conditions. a) DCM:ETA (50:50%) (2ml), MeOH (2 ml), H_2O (2 ml)), load plasma +100ng/mL PAHs+100ng/mL P.D10 (1 ml),wash 2 ml H_2O, elution DCM:ETA (50:50%) (2 ml); b) DCM (2ml), MeOH (2 ml), H_2O (2 ml)), load plasma +100ng/mL PAHs+100ng/mL P.D10 (1 ml)+9 ml MeOH:H_2O (10:90%) ,wash 2 ml H_2O, elution DCM:ETA (50:50%) (2 ml); c) DCM (6ml), MeOH (6 ml), H_2O (6 ml)), load plasma +100ng/mL PAHs+100ng/mL P.D10 (1 ml)+19 ml MeOH:H_2O (10:90%) ,wash 12 ml H_2O, elution DCM:ETA (50:50%) (5 ml). Elution order: 1-N, 2-Ace, 3-Fl, 4-P, 5-P.D10, 6-A, 7-Flu, 8-Py, 9-BaA, 10-Ch, 11-B(b)Flu, 12-B(k)Flu, 13-BaPy, 14-D(ah)A 15-BghiPY, 16-IPy.

Table S1. Linearity parameters

PAHs	Calibration curves	R^2	Sy	F value	Slope p-value	Tstat
**N**	202.33x+1.535	0.9997	1.14	555112	1.97277E-07	74.24
**Ace**	92.032x+0.116	0.9991	1.26	4527.3	2.92309E-07	67.28
**Fl**	190.62x-0.318	0.9997	0.77	12115	4.08576E-08	110.1
**P**	105.75x+1.943	0.9990	1.31	4155.8	3.46846E-07	64.47

**A**	33.875x+0.737	0.9993	1.14	5460.0	2.0102E-07	73.89
**Flu**	172.77x+1.238	0.9983	1.75	2336.4	1.09599E-06	48.34
**Py**	194.02x+0.856	0.9972	2.25	1410.3	3.00261E-06	37.55
**BaA**	59.139x+0.310	0.9997	0.79	11609	4.44939E-08	107.7
**Ch**	156.14x+0.178	0.9993	1.12	5741.8	1.8178E-07	75.77
**B(b)Flu**	183.86x+1.432	0.9989	1.41	3628.4	4.54901E-07	60.24
**B(k)Flu**	76.292x+0.523	0.9998	0.58	21271	1.32572E-08	145.8
**BaPy**	81.132x+0.575	0.9996	0.86	9616.4	6.48378E-08	98.06
**D(ah)A**	285.86x+0.527	0.9971	2.27	1393.5	3.07517E-06	37.33
**BghiPY**	154.18x+0.871	0.9994	1.04	6686.4	1.34071E-07	81.77
**IPy**	290.79x+1.062	0.9992	1.23	4762.5	2.64166E-07	69.01

Table S2. Stability of retention time and estimated concentrations using regression slopes.

**PAHs**	**Rt average**	**RSD%**	**Estimated concentration**	**RSD%**
N	3.2	1.23	100.1	0.56
Ace	5.65	1.04	100.2	0.45
FL	6.385	1.20	100.0	0.26
P	8.26	0.49	98.9	1.10
A	10.055	1.13	99.1	1.25
Flu	11.485	0.19	100.7	0.85
Py	11.98	0.09	100.5	0.56
BA	13.805	0.08	99.3	0.92
Ch	14.045	0.07	99.1	1.02
BbFlu	15.145	0.13	100.1	0.26
BkFlu	15.58	0.17	98.9	0.86
BaPy	15.98	0.18	99.1	0.72
DahA	17.025	0.24	99.6	1.25
BghiP	17.605	0.27	100.1	0.93
IPy	18.195	0.38	100.5	0.25
